# Reduced chemotherapeutic sensitivity in high glucose condition: implication of antioxidant response

**DOI:** 10.18632/oncotarget.27087

**Published:** 2019-07-23

**Authors:** Alessia Garufi, Gianandrea Traversi, Maria Saveria Gilardini Montani, Valerio D’Orazi, Giuseppa Pistritto, Mara Cirone, Gabriella D’Orazi

**Affiliations:** ^1^IRCCS Regina Elena National Cancer Institute, Department of Research, Rome 00144, Italy; ^2^University ‘G. d’Annunzio’, Department of Medical and Biotechnological Sciences, Chieti 66013, Italy; ^3^Sapienza University, Department of Experimental Medicine, Rome 00161, Italy; ^4^Department of Surgical Sciences, Rome 00161, Italy; ^5^University Tor Vergata, Department of Systems Medicine, Rome 00133, Italy; ^*^These authors contributed equally to this work

**Keywords:** chemoresistance, reactive oxygen species (ROS), high glucose (HG), cancer, nuclear factor erythroid 2-related factor 2 (NRF2)

## Abstract

Resistance to chemotherapy represents a major obstacle to successful treatment. The generation of reactive oxygen species (ROS) has been directly linked to the cytotoxic effects of several antitumor agents, including Adriamycin (ADR), and modulation of the oxidative balance has been implicated in the development and/or regulation of resistance to chemotherapeutic drugs. We recently showed that high glucose (HG) markedly diminished the cancer cell death induced by anticancer agents such as ADR. In the present study we attempted to evaluate the mechanism that impaired the cytotoxic effect of ADR in HG. We found that, in colon cancer cells, HG attenuated ADR-induced ROS production that consequently diminished ADR-induced H2AX phosphorylation and micronuclei (MN) formation. Mechanistically, HG attenuation of ADR-induced ROS production correlated with increased antioxidant response promoted by NRF2 activity. Thus, pharmacologic inhibition of NRF2 pathway by brusatol re-established the ADR cytotoxic effect impaired by HG. Together, the data provide new insights into chemotherapeutic-resistance mechanisms in HG condition dictated by increased NRF2-induced antioxidant response and how they may be overcome in order to restore chemosensitivity and ADR-induced cell death.

## INTRODUCTION

Chemotherapy can kill the drug-sensitive cancer cells, but the drug-resistant cells left behind can cause tumor recurrence (or relapse) and even cancer metastasis [1]. Development of drug resistance is therefore an important factor in anticancer therapeutic failure [2]. Two types of resistance occur, that is, intrinsic or acquired. Mechanistically, a variety of different systems contribute to chemoresistance including tumor heterogeneity, drug-inactivation, evasion of apoptosis, enhanced DNA repair and increased drug efflux [3–5]. The effectiveness of traditional cancer chemotherapy is largely based on the generation of reactive oxygen species (ROS) and consequently on the oxidative stress that exceeds the reduction capacity of cancer cells, leading ultimately to apoptosis or necrosis [6–8]. Most conventional and also non conventional chemotherapeutic agents as well as radiotherapeutic agents kill cancer cells by stimulating ROS generation [9–12]. Redox resetting usually occurs in anticancer drug treatment as a protective response from tumor cells to cope with drug-induced stress and DNA damage, leading ultimately to drug resistance [13]. Thus, alteration in redox balance, and deregulated redox signaling are common hallmarks of cancer progression and resistance to treatment [14]. ROS balance is typically regulated by antioxidant enzymes including catalase, superoxide dismutase (SOD) and glutathione S-transferase (GST) which detoxify ROS, reduce ROS-dependent apoptosis and attenuate chemotherapeutic cytotoxicity [8, 10]. Antioxidant protein expression is regulated by the transcription factor nuclear factor erythroid 2-related factor 2 (NRF2) which is the major regulator of the antioxidant response [15]. NRF2 is activated during oxidative and electrophilic stress through release from its inhibitory Keap1 (Kelch-like ECH-associated protein 1) to bind antioxidant responsive elements (ARE) in the promoter of target genes including catalase, GST, SOD, and NAD(P)H quinone oxidoreductase 1 (NQO1) [16], promoting their transcription. However, noncanonical activation of NRF2 may also occur and is mediated by p62-induced KEAP1 degradation through autophagy [17] or by the p53 target p21 which up-regulates the NRF2 signaling pathway by interrupting KEAP1/NRF2 interaction and therefore inducing NRF2 stabilization [18]. Activation of the NRF2-induced pathway in cancer has been shown to be critical for chemotherapeutic resistance. Among the NRF2 targets, catalase overexpression has been shown to protect cancer cells from apoptosis induced by DNA-damaging agents [19, 20]. Similarly, a role of NQO1 in chemotherapeutic resistance has been demonstrated and inhibition of NQO1 has been shown to suppress cancer cell growth and to potentiate cytotoxicity of anticancer agents [21–23]. Therefore, targeting NRF2 signaling may be a potentially attractive target to combat chemoresistance [15, 16, 24]. Among NRF2-targeting agents brusatol, a quassinoid extracted from *Brucea javanica*, that has been shown to enhance the efficacy of chemotherapy by inhibiting the NRF2-mediated defense mechanism [25, 26].

We have previously reported that attenuation of drug-induced cancer cell death occurs when cancer cells are switched form low to high glucose (HG) condition and are treated with Adiamycin, (ADR), highlighting by several mechanisms [27–30]. Since the production of ROS induced by the chemotherapeutic drug ADR is considered a major trigger for apoptotic cell death [31], in the present study we aimed at evaluating whether the reduced chemotherapeutic sensitivity promoted by HG in colon cancer cells might depend on deregulated ADR-induced ROS generation. To this end we evaluated the production of ROS and of the antioxidant response induced by ADR under conditions of low and high glucose in colon cancer cell lines, *in vitro*, and how it can modify drug-induced cell death.

## RESULTS

### High glucose (HG) attenuated the ADR-induced ROS generation

**Figure 1:**
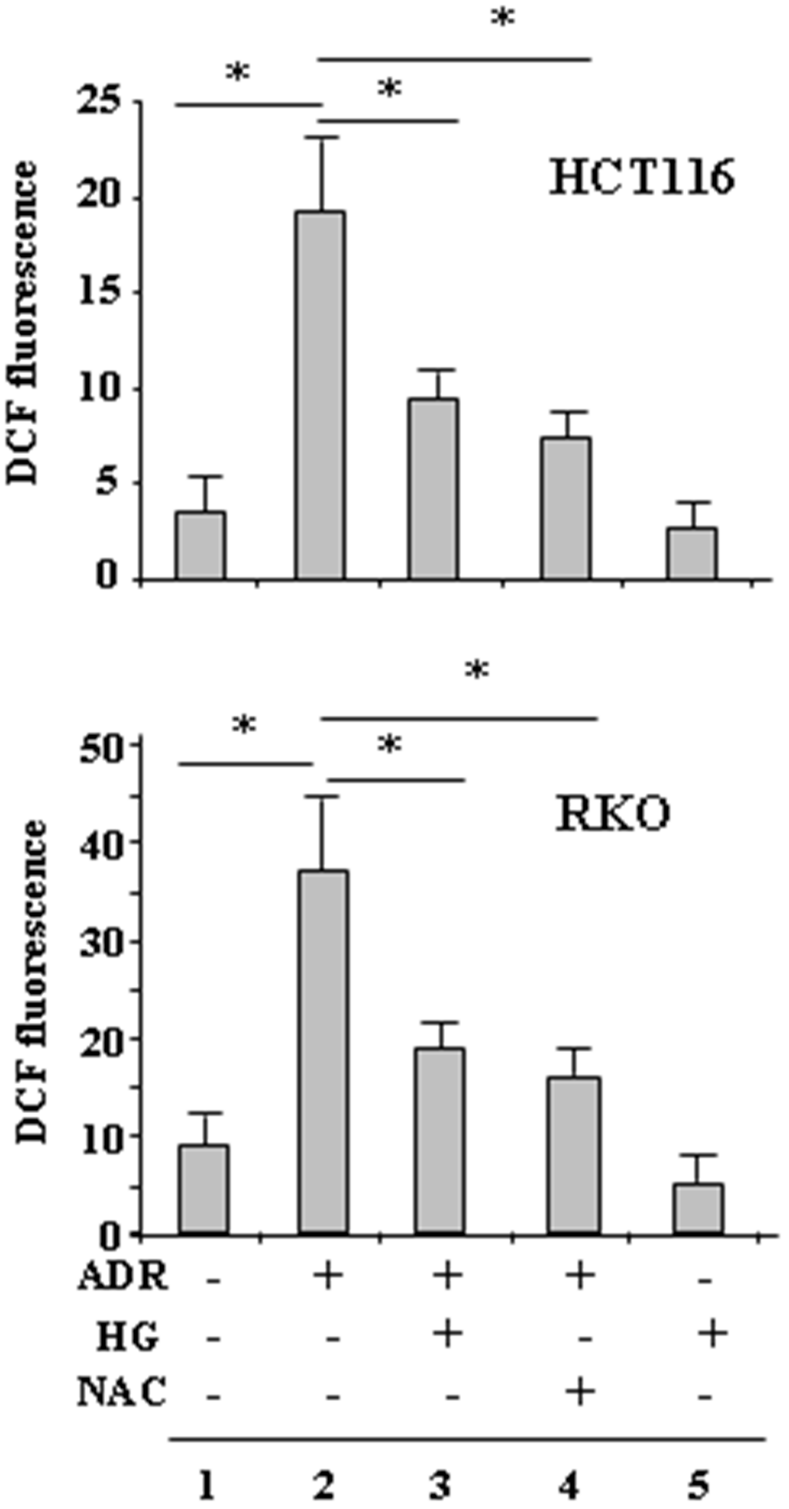
HG reduces ADR-induced ROS generation. HCT116 and RKO were treated with ADR (2 μg/ml for 6 h) in low or high glucose (HG) with or without NAC (10 *μ*M, added 1 h before ADR). Then, the cells were incubated with 10 μM DCFDA and fluorescence was determined by flow cytometry. Data shown are the means ± s.d. of *n* = 3 independent experiments; ANOVA test with Bonferroni correction: ^*^ *p* < 0.001.

The ROS levels were first assessed in RKO and HCT116 cells treated with ADR in low and HG condition, by DCF fluorescence. Results show that ROS levels markedly increased when the cells were treated with ADR in low glucose (Figure 1, compare lane 2 with lane 1) but not when they were treated with ADR in HG (Figure 1, compare lane 2 with lane 3). Attenuation of ADR-induced ROS in HG was comparable to that obtained by using the ROS scavenger NAC in low glucose (Figure 1, compare lane 2 with lane 3 and lane 2 with lane 4). Of note, HG per se did not modify intracellular ROS levels, indicating that it was instead triggering some mechanisms to reduce the effect of the drug. These data indicate that HG lowered the production of ROS induced by ADR to levels comparable to that obtained by NAC inhibition of ROS production by ADR in low glucose.

### High glucose (HG) reduced ADR-induced DNA damage and cell death

**Figure 2:**
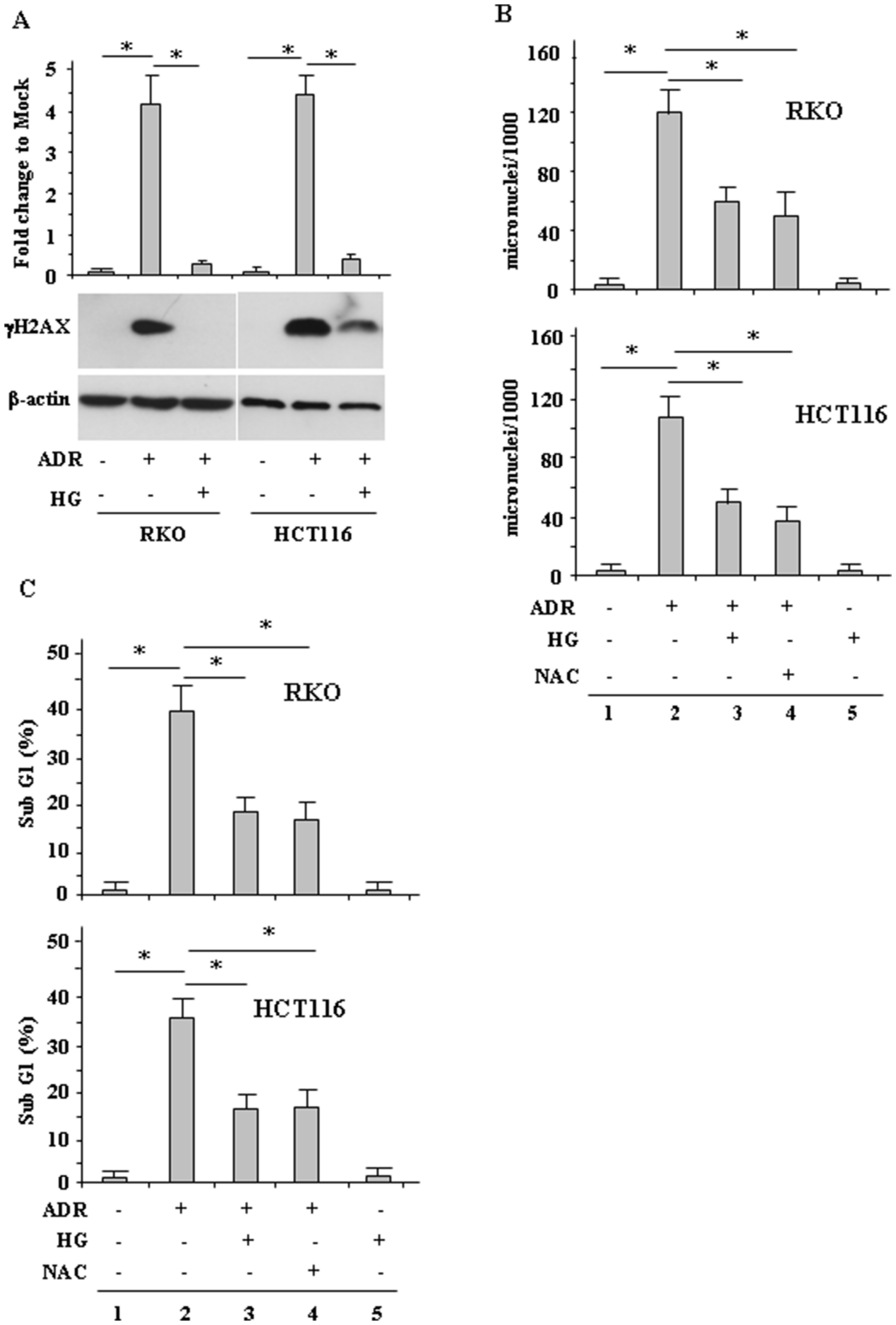
HG reduces ADR-induced DNA damage. (**A**), RKO and HCT116 were treated with ADR (2 μg/ml for 16 h) in low or high glucose (HG). Equal amount of total cell extracts was analysed by Western immunoblotting with anti-*γ*H2AX antibody. Representative images are shown. Anti-*β*-actin was used as protein loading control. Densitometric analysis was applied to quantify *γ*H2AX expression/*β*-actin ratio and expressed as fold change to Mock (upper panel). Data are presented as the means ± s.d. of *n* = 4 independent experiments; ANOVA test with Bonferroni correction: ^*^ *p* < 0.001. (**B**), RKO and HCT116 cells were treated with ADR (2 μg/ml) for 1 h with or without 1 h pretreatment with NAC (10 μM), in low and HG. After treatments, fresh medium was added with 1 μg/ml cytochalasin B for 48 h in order to obtain binucleated (BN) cells. Then, cells were stained with Giemsa and observed under a light microscope using high magnification (×1000). The results are expressed as total MN on 1000 BN cells (MN‰). Data are presented as the means ± s.d. of *n* = 3 independent experiments; ANOVA test with Bonferroni correction: ^*^ *p* < 0.001. (**C**), RKO and HCT116 cells were treated with ADR (2 μg/ml) in low and HG with or without 1 h pretreatment with NAC (10 μM). After 24 h treatment, cells were fixed and stained with PI for sub-G1 evaluation. Data are presented as the means ± s.d. of *n* = 3 independent experiments. ANOVA test with Bonferroni correction: ^*^ *p* < 0.001.

Then, we evaluated whether the modulation of ROS had an effect on drug-induced DNA damage and consequently on cell death. To this aim, γH2AX levels and micronuclei (MN) formation were evaluated. H2AX phosphorylation in Ser139, generating γH2AX, occurs in general in response to double-strand brakes (DSB) and is an early sign of replication stalling [32]. We found that ADR treatment in low glucose strongly induced γH2AX levels while ADR treatment in HG condition failed to do so (Figure 2A), suggesting reduction of DNA damage. MN are acentric chromosomal fragments or whole chromosomes lost during cell division as a result of DNA damage and are commonly detected in cells exhibiting intrinsic genomic instability or following exposure to genotoxic agents [33, 34]. We found that the ADR-induced MN formation in low glucose (Figure 2B, compare lane 2 with lane 1) was significantly reduced in HG condition (Figure 2B, compare lane 2 with lane 3). To evaluate the role of ROS in MN formation, the ROS scavenger NAC was used. The results show that the ADR-induced MN formation in low glucose was greatly reduced by NAC treatment (Figure 2B, compare lane 2 with lane 4) to the levels obtained by ADR in HG (Figure 2B, compare lane 4 with lane 3), confirming the role of drug-induced ROS in DNA damage.

Since drug-induced ROS generation and DNA damage are the major trigger for cell death [9–11] the cell viability was evaluated by FACS analysis. As shown in Figure 2C, ADR treatment markedly increased cell death in low glucose (Figure 2C, compare lane 2 with lane 1), but this effect was attenuated by HG (Figure 2C, compare lane 2 with lane 3). In addition, NAC reduced ADR-induced cell death in low glucose (Figure 2C, compare lane 4 with lane 2) to the levels obtained by ADR in HG (Figure 2C, compare lane 4 with lane 3), in agreement with the above role of drug-induced ROS in inducing DNA damage. Of note, HG alone did not change cell death (Figure 2C), as previously shown [27]. Together, these results indicate that HG reduced the ADR-induced cell death as a consequence of attenuation of ROS production.

### High glucose (HG) increased antioxidant response during ADR treatment

**Figure 3:**
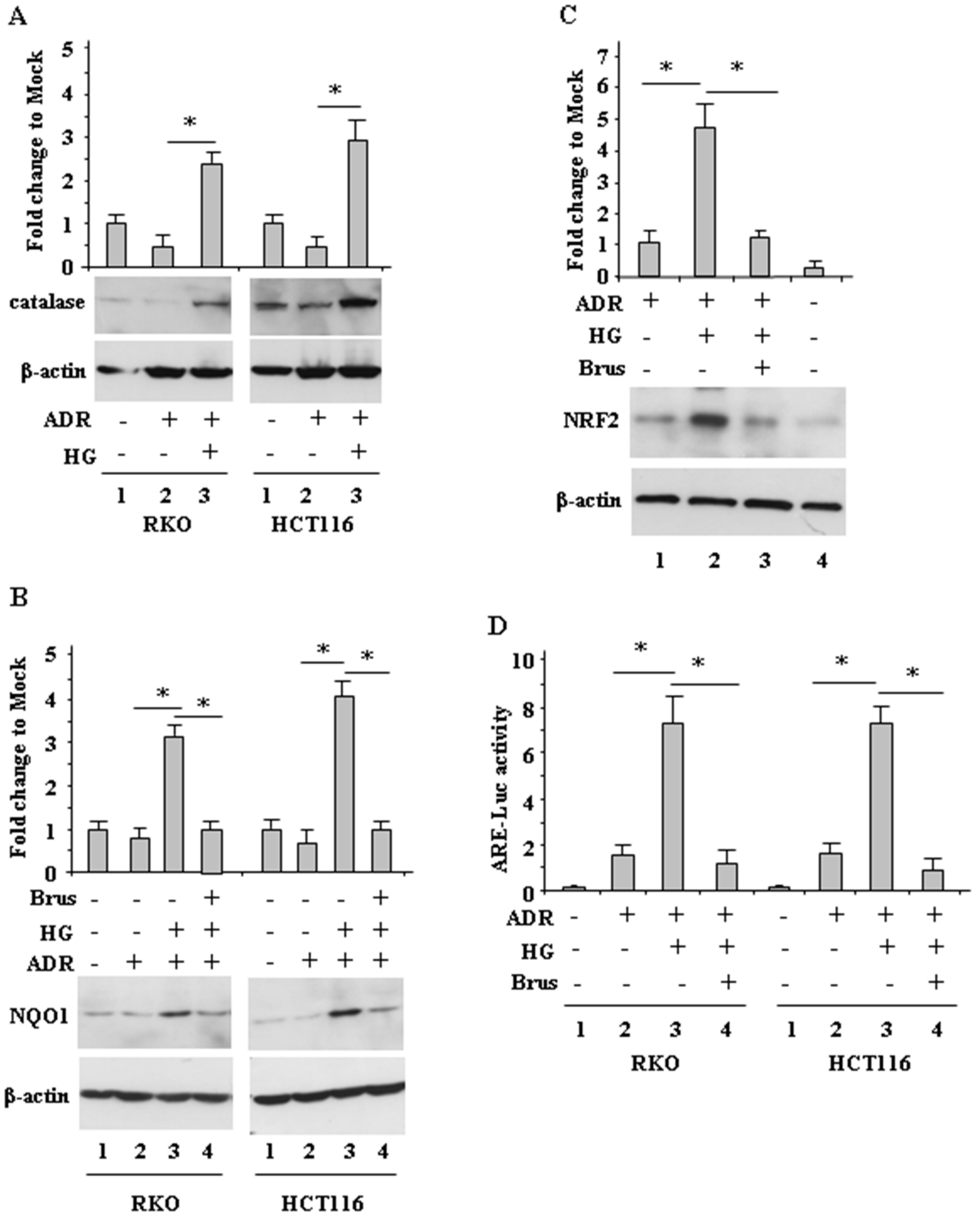
Increased antioxidant response during ADR treatment in HG condition. (**A**), RKO and HCT116 cells were treated with ADR (2 μg/ml for 16 h) in low glucose or HG. Equal amount of total cell extracts was analysed by Western immunoblotting with anti-catalase antibody. Representative images are shown. Anti-β-actin was used as protein loading control. Densitometric analysis was applied to quantify catalase expression/β-actin ratio and expressed as fold change to Mock, fixing the control to 1. (**B**), RKO and HCT116 cells were pre-treated with brusatol (100 nM for 4 h) and then treated with ADR (2 μg/ml for 16 h) in low glucose or HG. Equal amount of total cell extracts was analysed by Western immunoblotting with anti-NQO1 antibody. Representative images are shown. Anti-β-actin was used as protein loading control. Densitometric analysis was applied to quantify NQO1 expression/β-actin ratio is expressed and expressed as fold change to Mock, fixing the control to 1. (**C**), RKO cells were pre-treated with brusatol (100 nM for 4 h) and then treated with ADR (2 μg/ml for 16 h) in low glucose or HG. Equal amount of total cell extracts was analysed by Western immunoblotting with anti-NRF2 antibody. Representative images are shown (upper panel). Anti-β-actin was used as protein loading control. Densitometric analysis was applied to quantify NRF2 expression/β-actin ratio and expressed as fold change to Mock, fixing the control to 1. (**D**), RKO and HCT116 cells were transfected with the NRF2 ARE-Luc vector and treated as in (C); then relative luciferase activity was measured. The results of ARE-luc activity are shown as the means ± s.d. of *n* = 3 independent experiments. ANOVA test with Bonferroni correction: ^*^ *p* < 0.001.

We next aimed at evaluating the mechanisms involved in ROS modulation by HG, keeping in mind that the antioxidant response contributes to chemotherapeutic-resistance [6, 9, 10, 14]. We found that the levels of catalase, a ROS scavenger enzyme [19] (Figure 3A) and of NQO1, another antioxidant protein (Figure 3B) markedly increased when cells were treated with ADR in HG, but not when cells were treated in low glucose (Figure 3A and Figure 3B, compare lane 3 with lane 2). In addition, greater NRF2 accumulation was achieved in cells treated with ADR in HG, compared to the treatment in low glucose (Figure 3C, compare lane 2 with lane 1). Since the modulation of NRF2 levels does not clearly indicate its function, the NRF2 transcriptional activity was assessed by the NRF2 ARE-Luc assay. As shown in Figure 3D, ARE-Luc activity was greatly increased by ADR in HG condition (Figure 3D, compare lane 3 with lane 2) but only slightly enhanced in low glucose condition (Figure 3D, compare lane 1 with lane 2). Next we aimed at evaluating the role of NRF2 in modulation of the antioxidant response, by its pharmacologic inhibition. Among the NRF2-targeting agents, brusatol, a quassinoid extracted from *Brucea javanica*, has been shown to inhibit the NRF2-mediated defense mechanism [25]. We found that the NQO1 levels (Figure 3B, compare lane 3 with lane 4) as well as the NRF2 levels (Figure 3C, compare lane 2 with lane 3) in ADR/HG were greatly reduced by brusatol. In agreement, brusatol markedly reduced ADR-induced NRF2 ARE-Luc activity in HG condition (Figure 3D, compare lane 3 with lane 4). These findings suggest that in ADR/HG condition an increased antioxidant response occurred, mediated by NRF2 activity.

### Inhibition of the antioxidant response rescued ADR-induced DNA damage and cell death, attenuated by high glucose (HG)

**Figure 4:**
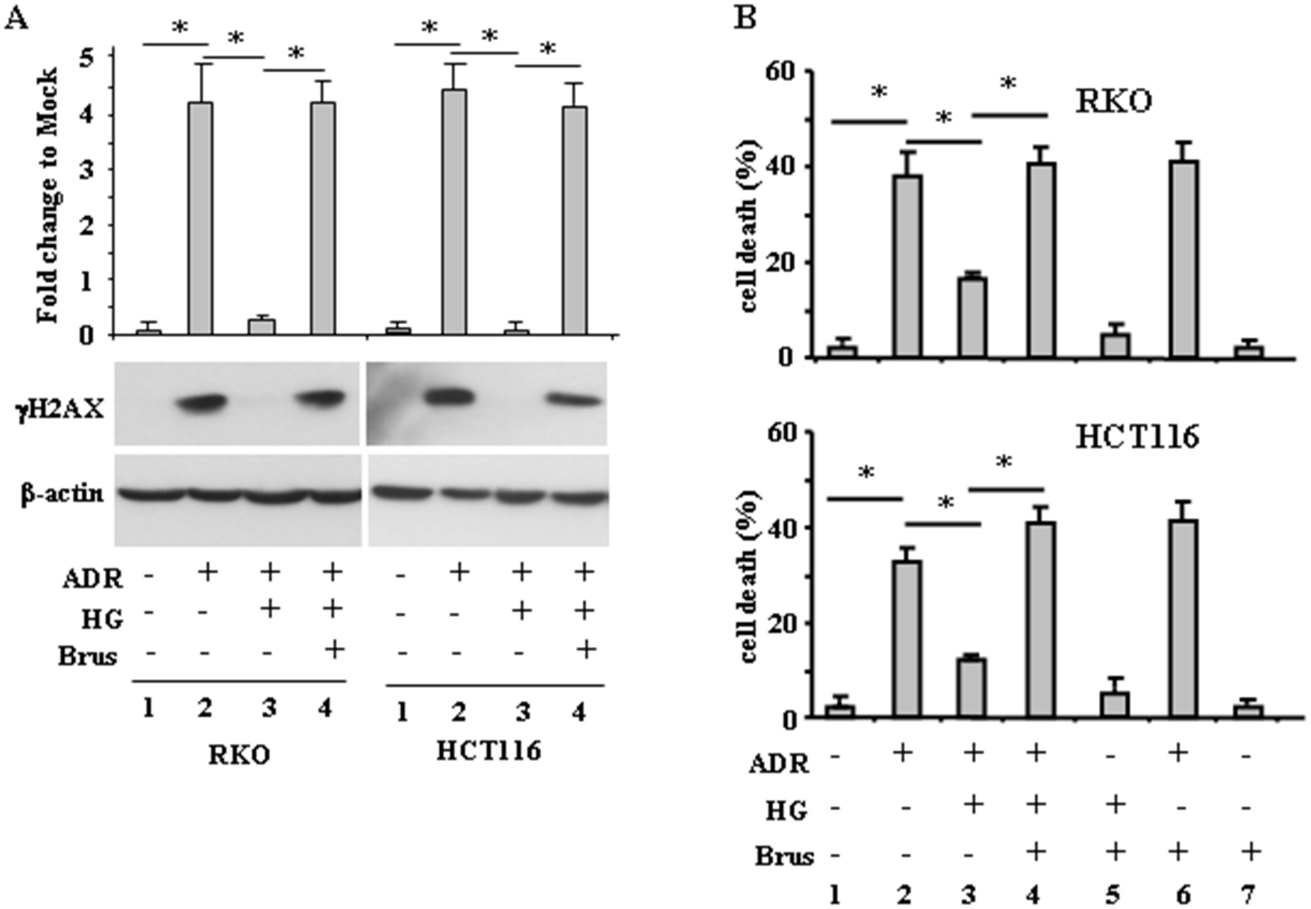
Brusatol restores ADR-induced DNA damage and cell death impaired by HG. (**A**), RKO and HCT116 cells were pre-treatred with brusatol (100 nM for 4 h) prior to adding ADR (2 *μ*g/ml for 16 h), in low glucose and in HG. Equal amount of total cell extracts was analysed by Western immunoblotting with anti-γH2AX antibody. Representative images are shown. Anti-*β*-actin was used as protein loading control. Densitometric analysis was applied to quantify NRF2 expression/*β*-actin ratio. (**B**), RKO and HCT116 cells were treated as in (A) and 24 h after treatments, the percentage of dead cells was scored by Trypan blue staining. Data shown are the means ± s.d. of *n* = 3 independent experiments. ANOVA test with Bonferroni correction: ^*^ *p* < 0.001.

Having established that an increased antioxidant response occurred when cells were treated with ADR in HG, we aimed at evaluating whether its targeting could rescue ADR-induced DNA damage and cell death, attenuated by HG. We found that the lack of H2AX phosphorylation in ADR/HG was counteracted by brusatol (Figure 4A, compare lane 3 with lane 4), taking back the γH2AX levels to those achieved by ADR in low glucose (Figure 4A, compare lane 4 with lane 2), suggesting re-establishment of ADR-induced DNA damage in HG. In agreement, cell viability analysis shows that the attenuation of ADR-induced cell death in HG (Figure 4B, compare lane 2 with lane 3), was counteracted by brusatol (Figure 4B, compare lane 3 with lane 4), taking back the cell death levels to those achieved by ADR in low glucose (Figure 4B, compare lane 4 with lane 2), suggesting re-establishment of ADR-induced cell death in HG. These data indicate that targeting the antioxidant response in HG could restore DNA damage and cell death in response to ADR.

## DISCUSSION

A number of medical conditions can increase blood glucose concentration (hyperglycemia), including diabetes mellitus (DM), obesity, pancreatitis, chronic stress, and cancer [35, 36], and hyperglycemia has been shown to attenuate tumor therapeutic response and to confer resistance to chemotherapy-induced cell death [37, 38]. At molecular level, HG modulates various signaling pathways that control cancer cell proliferation, migration and recurrence [39, 40], as well as apoptosis [27, 39–43]. In our previous studies we showed that HG reduces ADR-induced cell death. Mechanistically, culturing cancer cells with HG or sera from diabetic patients reduces the drug-induced apoptotic activity of p53 [27, 28]. In this metabolic condition p53 changes its affinity for target promoters preferring for instance to transcribe *d*amage-*r*egulated *a*utophagy *m*odulato (DRAM) that induces autophagy, instead of transcribing *p*53 *u*pregulated *m*odulator of *a*poptosis (PUMA), inducing a pro-survival autophagy whose inhibition can re-establish drug cytotoxicity [29]. The HG condition inhibits also the drug-induced c-jun-N-terminal kinase (JNK) pathway that can be rescued by ZnCl_2_ supplementation that counteracts the glycolytic pro-survival pathway restoring cancer cell chemosensitivity [30]. *In vivo* study confirmed that ADR treatment does not reduce the growth of a xenograft tumor in diabetic mice, as it does in normal glycemic control mice [29]. These findings are in agreement with preclinical studies where hyperglycemia is associated with attenuation of the antiproliferative effect of chemotherapy [44], underlining a multifaceted influence of hyperglycemia in the regulation of cancer cell chemosensitivity.

Most conventional and also non conventional chemotherapeutic agents as well as radiotherapeutic agents kill cancer cells by stimulating ROS generation that promotes DNA damage [6, 9–12, 14]. In agreement, here we found that ADR-induced ROS increased DNA damage ultimately inducing cell death. However, when ADR-induced ROS production was attenuated by HG, also H2AX phosphorylation and MN formation were reduced, along with cell death, linking the ROS production with DNA damage and cell death and therefore with the cell ability to cope with oxidative stress in order to survive. Therefore, we hypothesized a role for the antioxidant response to explain ROS modulation in ADR/HG condition, corroborated by the fact that persistent oxidative stress induces adaptive responses including antioxidant up-regulation conferring resistance to apoptosis [7]. Thus, we found increased catalase and NQO1 levels in ADR/HG, suggesting indeed increased antioxidant response. Catalase overexpression has been shown to protect cancer cells from apoptosis induced by DNA-damaging agents, rendering catalase a future therapeutic target [19, 20]. Similarly, a role of NQO1 in cancer chemotherapy has been demonstrated by several groups and inhibition of NQO1 has been shown to suppress cancer cell growth and to potentiate cytotoxicity of anticancer agents [21–23] and its downregulation may restore cancer cell sensitivity to chemotherapeutic agents as an attractive strategy for treating cancers. Although the antioxidant response can be seen as an obstacle to the drug cytotoxic effect, one limitation of our study is that we used only one drug to show the antioxidant mechanism in HG condition. It would be interesting to test if also other drugs that stimulate ROS generation as a mechanism of cell death might undergo increased antioxidant response in HG. Therefore, more experiments with different drugs should be performed in order to generalize these findings and make them useful for clinical applications. Another point that needs to be further explored is if high glucose could interfere with uptake of ADR or be direct on adriamycin metabolism by impairing the effectiveness of the chemotherapy itself.

Antioxidant proteins expression is regulated by NRF2 whose activation provides a growth advantage for cancer cells, protects against oxidative stress, and contributes to chemotherapeutic-resistance [45, 46]. Under unstressed condition, NRF2 forms a complex with Keap1, leading to protein degradation. Upon exposure to different stressors, including ROS, toxic agents and carcinogens, NRF2 is released from Keap1, translocates to the nucleus, binds AREs in antioxidant gene promoters and up-regulates expression of target genes [47]. However, noncanonical activation of NRF2 may exists mediated, for instance, by p53 target p21 that, by interrupting KEAP1/NRF2 interaction, induces NRF2 accumulation and up-regulates the NRF2 signaling pathway [18]. Here, we found that ADR treatment in HG condition increased the NRF2 activity and the expression of NRF2 target genes catalase and NQO1, thus contributing to reduce the drug cytotoxic effect. In the attempt to explain the mechanisms of NRF2 up-regulation, we can argue that p53 may play a role. Thus, we have previously reported that HG, by modulating p53 post-translational modifications and therefore p53 transactivation function, reduces the p53-induced apoptotic gene transcription while increases the transcription of autophagy gene such as DRAM or p21 (data not shown). It could be likely that p21, in this ADR/HG setting, could activate NRF2, although further studies are needed to explain this link.

Having assessed that an increased antioxidant response occurred in ADR/HG, we aimed at evaluating whether its manipulation could rescue ADR-induced DNA damage and cell death, attenuated by HG. NRF2-targeting agents can be used to overcome antioxidant response-dependent chemotherapeutic-resistance [24] and brusatol, a quassinoid extracted from *Brucea javanica*, has been shown to enhance the efficacy of chemotherapy by inhibiting the NRF2-mediated defense mechanism [25, 26]. Brusatol may abrogate gemcitabine-induced NRF2 activation in pancreatic cancer cells to restore chemosensitivity of cancer cells [48]; may enhance the radiosensitivity of lung cancer cells by promoting ROS production and enhancing DNA damage [49]; and may inhibit cancer cell growth and induce apoptosis via JNK/p38 MAPK/NF-κb/Stat3/Bcl-2 [50]. In line with these findings, we show that brusatol re-sensitized cancer cells to drug cytotoxic activity inhibited by HG and inhibited NRF2-dependent activity.

In summary, the present study shows that HG reduced ADR-induced cell death by impairment of ROS production and increased antioxidant response through induction of the NRF2 activity. Therefore, this study may suggest that targeting the antioxidant response might contribute to restore chemotherapeutic sensitivity and ADR-induced cell death, inhibited by HG. In addition, once this mechanism is validated by using different anticancer agents, the concept to highlight the antioxidant response could be exploited also for predictive and prognostic purposes. To this aim, the new technological method of liquid biopsy could be of help in measuring for instance circulating tumor DNA and micro-RNA modified by HG and in response to drugs [51–53].

## MATERIALS AND METHODS

### Cell culture and reagents

Mycoplasma negative human RKO and HCT116 colon cancer cell lines (ATCC) were routinely cultured in Dulbecco modified Eagle’s medium (DMEM) (Life Technology-Invitrogen, Carlsbad, CA, USA) containing 1 g/L D-glucose (considered low glucose) supplemented with 10% heat-inactivated foetal bovine serum (FBS) (GIBCO-BRL, Grand Island, NY, USA) plus 100 units/ml penicillin/streptomycin and glutamine in 5% CO_2_ humidified incubator at 37°C. For the experiments in high glucose (HG), cells, routinely cultured in medium containing 1 g/L D-glucose, were washed in PBS, and then replaced with culture medium containing 4.5 g/L D-glucose (high glucose – HG) plus 2% FBS, as previously reported [27, 39, 40]. Control experiments in low glucose were also performed in 2% FBS.

The chemotherapeutic drugs Doxorubicin (herein referred as Adriamycin, ADR) (Sigma-Aldrich) was added to the culture media at 2 μg/ml for the indicated times. The ADR amount used in this study, that is 2 μg/ml, was previously reported by us to induce apoptosis in RKO and HCT116 cells [27]. The ROS inhibitor N-acetyl-L-cysteine (NAC) (Sigma-Aldrich, St Louis. MO, USA) was used at 10 μM; antioxidant response inhibitor Brusatol (Sigma-Aldrich) was used at 100 nM; Cytochalasin B (Sigma, St Louis, MO, USA) was used at 1 μg/ml.

### Measurement of intracellular reactive oxygen species (ROS) production

To measure reactive oxygen species (ROS) production, we used 2′,7′-dichlorofluorescein diacetate (DCFDA; Sigma–Aldrich), a highly fluorescent compound that after diffusion into the cell, is oxidized by ROS into 2′,7′-dichlorofluorescein (DCF), and can be detected by fluorescence spectroscopy, as reported [54]. Briefly, cells were plated in duplicate on six-well culture plates. The day after plating, cells were washed in PBS before adding HG culture media and low glucose media for the controls, for 24 h prior to adding the indicated treatments (ADR - 2 μg/ml - for 6 h in the presence or absence of NAC - 10 μM - added 1 h before ADR). Following treatment, cells were washed with PBS and incubated with 10 μM DCFDA, for 15 min at 37°C. Cell pellets were then collected and analyzed in the FL-1 channel of a FACScalibur flow cytometer (Becton–Dickinson). DCF fluorescence was determined using 5 × 10^5^ cells/ml and values expressed as mean DCF fluorescence per cell population.

### Cell viability

To measure cell viability we used Trypan blues staining, as previously reported [27]. Briefly, equal numbers of cells were plated in duplicate in 60 mm Petri dishes. The day after plating, cells were washed in PBS before adding HG culture media and low glucose media for the controls, for 24 h before dispensing the indicated treatments. Cell viability was determined by Trypan blue staining of both floating and adherent cells and was measured by direct counting blue/white cells with a haemocytometer. The percentage of dead cell (blue/total) was calculated from 200 cells per well in triplicate.

### Cell death/PI staining

Cell death was quantified by Fluorescence Activated Cell Sorting (FACS) analysis, staining cells with the nonvital dye propidium iodide (PI) (Immunological Sciences, Rome, Italy), following the manufacturer’s instruction. Briefly, adherent cells were tripsinized and then collected along with floating cells by centrifugation; then, cell pellets were fixed in 80% ethanol and stained in a PBS solution containing propidium iodide (PI) (62.5 mg/ml; Sigma-Aldrich) and RNase A Q10 (1.125 mg/ml; Sigma-Aldrich), as reported [55]. Stained samples were then analyzed with a FACScan instrument (Becton Dickinson Europe Holdings SAS - Le Pont De Claix, France) and the percentage of cells in sub G1 compartment was calculated using ModFit LT software (Becton Dickinson). Approximately 30000 events were acquired and gated using forward scatter and side scatter to exclude cell debris.

### Analysis of micronuclei (MN) by Cytokinesis-block micronucleus (CBMN) assay

Analysis of MN was assessed by Cytokinesis-block micronucleus (CBMN) assay [56]. Briefly, 3 × 10^5^ cells were seeded on coverslips in 60 mm Petri dish. The day after plating, cells were washed in PBS before adding HG culture media and low glucose media for the controls, for 24 h prior to adding a pulse of ADR (2 μg/ml) for 1 h, with or without NAC (10 μM, added 1 h before ADR). Following treatment, cells were replaced with fresh medium containing 1 μg/ml cytochalasin B, for 48 h, in order to obtain binucleated (BN) cells. Cells were then fixed in cold methanol for 10 min, air dried and stained with Giemsa. The coverslips were mounted on slides that were observed under a light microscope using high magnification (×1000). For each experimental point, at least 1000 BN cells were analysed, cells with one or more MN recorded and results expressed as total MN on 1000 BN cells (MN‰).

### Western blot analysis

Total cell extracts were prepared by incubation in lysis buffer (50 mM Tris-HCl, pH 7.5, 150 mM NaCl, 5 mM EDTA, pH 8.0, 150 mM KCl, 1 mM dithiothreitol, 1% Nonidet P-40) and a mix of protease and phosphatase inhibitors (Roche, Indianapolis, IN, USA) on ice for 30 min. Cell debris was removed by centrifugation (15000 × g for 20 min) and supernatant collected. Protein concentration was determined using BCA Protein Assay kit (BioRad, Hercules, CA, USA). Samples were denatured in SDS sample buffer. Total cell extracts (10–40 μg total cell lysate/lane) were resolved by 9–18% SDS polyacrylamide gel electrophoresis and transferred to polyvinylidene difluoride (PVDF) (Merck Millipore, Billerica, MA, USA) or nitrocellulose (BioRad) membranes by using the MiniPROTEAN 3 apparatus (Bio-Rad). Unspecific binding sites were blocked by incubating membranes for 1 h in 0.05% Tween-20 (v/v in TBS) supplemented with 5% non-fat powdered milk or bovine serum albumin (BSA) (SIGMA-Aldrich), followed by overnight (o/n) incubation with the following primary antibodies: mouse monoclonal anti-phospho-Histone H2AX (Ser139) (Millipore), rabbit monoclonal anti-NRF2 (Abcam, ab62352), mouse monoclonal anti-catalase (H-9) (Santa Cruz Biotechnology, sc-271803), mouse monoclonal anti-NQO1 (A180) (Thermo-Scientific). Primary antibodies were detected with appropriate anti-immunoglobulin-G-horseradish peroxidase secondary antibodies (BioRad). Enzymatic signals were visualized by chemiluminescence (ECL Detection system, Amersham GE Healthcare, Milan, Italy), according to the manufacturer’s protocol. Equal lane loading was monitored by probing membranes with antibodies specific for mouse monoclonal β-actin (Calbiochem, San Diego, CA, USA). Densitometry was performed with ImageJ software and relative band intensity normalized to β-actin and quantified with respect to controls set to 1.0.

### NRF2 ARE-Luc activity

Sub-confluent cells were plated on white clear bottom 96-multiwell culture plates. The day after, cells were transfected with the NRF2 ARE-Luc reporter vector (ARE Reporter kit, antioxidant pathway, BPS Bioscience), using Lipofectamine Plus reagent according to the manufacturer’s instructions (Invitrogen, Carlsbad, CA, USA). Sixteen hours after transfection, cells were washed in PBS before adding HG culture media, and low glucose media for the controls, pre-treated with brusatol (100 nM for 4 h) followed by the addition of ADR (2 μg/ml for 16 h). Luciferase activity was assayed on whole-cell extracts, according to the manufacturer’s instructions.

### Statistical analysis

Each experiment, was performed at least three times. Results are reported as the mean ± standard deviation (s.d.) or as fold change to mock or as protein expression/β-actin ratio expressed as numbers underneath images fixing the control to 1. Statistical significance was determined using one-way ANOVA analysis for three or more sample comparisons using GraphPad Prism software (San Diego, CA, USA), with post hoc Bonferroni correction. A value of *p* < 0.05 was considered statistically significant.

## Abbreviations

ADR: adriamycin
ARE: antioxidant responsive elements
CBMN: cytokinesis-block micronucleus
DCF: dichlorofluorescein
DCFDA: dichlorofluorescein diacetate
DM: diabetes mellitus
DMEM: Dulbecco modified Eagle’s medium
DSB: double-strand brakes
FBS: foetal bovine serum
GST: glutathione S-transferase
HG: high glucose
HO-1: heme oxygenase 1
JNK: c-JunN-terminal kinase
Keap1: Kelch-like ECH-associated protein 1
MN: micronuclei
NAC: N-acetyl-L-cysteine
NQO1: NAD(P)H:quinone oxidoreductase 1
NRF2: nuclear factor erythroid 2-related factor 2
PI: propidium iodide
PVDF: polyvinylidene difluoride
ROS: reactive oxygen species
SD: standard deviation
SOD: superoxide dismutase
